# Knowledge and attitudes towards electroconvulsive therapy in an academic psychiatric department

**DOI:** 10.4102/sajpsychiatry.v30i0.2302

**Published:** 2024-11-21

**Authors:** Lerato L. Masenya, Yvette M. Nel

**Affiliations:** 1 Department of Psychiatry, Faculty of Health Sciences, University of the Witwatersrand, Johannesburg, South Africa

**Keywords:** electroconvulsive therapy, knowledge, attitudes, psychiatry, psychology, South Africa

## Abstract

**Background:**

Negative attitudes towards electroconvulsive therapy (ECT) among health professionals have been attributed to a lack of knowledge, despite advancements in its administration and evidence of its efficacy in certain psychiatric conditions.

**Aim:**

This study assesses knowledge and attitudes towards ECT among psychiatry and clinical psychology professionals.

**Setting:**

The University of the Witwatersrand, Department of Psychiatry, Johannesburg, South Africa.

**Methods:**

A quantitative cross-sectional design was used. All psychiatry and clinical psychology professionals associated with the university were invited to participate in an anonymous online survey from 01 September 2022 to 30 June 2023.

**Results:**

The response rate was 49.6% (*n* = 58) among psychiatrits and 22.2% (*n* = 22) among clinical psychology professionals. Psyhiatrists had greater knowldge and more favourable attitudes than psychologists. Only 45.5% of psychologists had exposure to ECT, compared to 93.1% of psychiatrists. Knowledge and attitude scores were significanly correlated (*p* = 0.009, OR 6.7). Most psychologists (86.4%) recommended ECT theory be included in their curriculum.

**Conclusion:**

Greater knowledge correlates with improved attitudes towards ECT. Increased training could enhance attitudes, particularly among psychology professionals.

**Contribution:**

This study offers insights into knowledge and attitudes towarss ECT in a Johannesburg psychiatry department.

## Introduction

Electroconvulsive therapy (ECT) is recommended as a first-line treatment where a rapid definitive response of depression is needed, where there is a high suicide risk, where there is severe psychomotor retardation associated with compromised eating and drinking, as well as to treat severe depression, mixed affective states, mania and catatonia.^1,2^ A systematic review investigating the effectiveness of ECT for the treatment of depression concluded that there have been no placebo-controlled randomised trials to justify its use since 1985. The author argues that the assessment of the efficacy of ECT should use placebo-controlled trials in keeping with standards applied to other medical interventions.^3,4^ Attitudes towards ECT have taken different forms throughout history, from concerns of long-bone fractures in the 1940s, questions of its relevance with introductions of effective psychopharmacological agents in the 1950s, to ongoing concerns about memory impairment and the proposed lack of quality evidence for use now in the 21st century.^3,4,5,6^

The use of ECT requires collaboration between psychiatrists who prescribe the procedure and anaesthesiologists who administer anaesthesia for the procedure. In addition to doctors, other mental health professionals play a role in decision making, patient education on ECT and referrals to psychiatrists for further interventions. In a study conducted locally, the majority of the multidisciplinary team members derived their knowledge of ECT from psychiatrists, with psychologists showing the lowest knowledge of the procedure.^7^ Psychiatrists often refer patients to clinical psychologists for a range of psychotherapeutic interventions, and clinical psychologists likewise refer to psychiatrists for medication and other interventions including ECT. For that reason, the rational, evidence-based use of ECT in the management of appropriately selected patients requires collaboration between the treating psychiatrist and clinical psychologist as part of a holistic multidisciplinary approach to care. In a literature review examining the psychological factors influencing patients’ responses to ECT, the author contends that clinical psychologists should play an active role in the consent process, utilise clinical formulation to comprehend patients’ perspectives and encourage patients to express their opinions about ECT to mental health professionals.^8^

Research into the knowledge of and attitudes towards ECT among healthcare professionals has been explored in various parts of the world; however, it is sparse in the South African context. A survey of the practice of ECT found that ECT was administered exclusively by consultant psychiatrists in South African private hospitals whereas most units in training hospitals used registrars to administer the procedure.^9^ Thus, the study of the knowledge of and attitudes towards ECT among psychiatry registrars may serve as an indicator of the likelihood of newer generations of psychiatry specialists prescribing ECT where indicated. Electroconvulsive therapy training is a requirement for specialisation in psychiatry as per the South African College of Psychiatrists. Formal training and exposure to ECT are not requirements for training in clinical psychology. However, the diagnosis of psychiatric and psychological disorders, including emergencies, and appropriate referral to other relevant professionals are a requirement for training and within the scope of practice for clinical psychology.^10,11,12^ The rationale for this study was, therefore, to provide a comparison of the knowledge of and attitudes towards ECT, a historically controversial procedure, between clinical psychologists and psychiatrists at a training institution in South Africa, with the view to provide insights to determine the direction of training on ECT.

## Research methods and design

### Study design and setting

This study is a quantitative cross-sectional online survey of psychiatrists, psychiatry registrars, community service and intern clinical psychologists, and clinical psychologists employed at various training hospitals falling under the University of the Witwatersrand’s Department of Psychiatry. Electroconvulsive therapy is an available treatment option at two hospitals within the university training circuit.

### Study population and sampling strategy

All psychiatry and clinical psychology professionals, 196 in total, were invited via email to participate in an online survey from 01 September 2022 to 30 June 2023. The invitation email contained a participation information sheet detailing the aim and objectives of the study, and a link to the consent form and the questionnaire. Voluntary response sampling was used. The questionnaire was initially live for 3 months, with a reminder email sent once a week yielding 59 responses (30.1%). The target response rate was 80%, with a minimum of at least 30% required to detect a large effect. The higher the response rate, the more likely that smaller significant differences between the groups can be detected; therefore, data collection was extended, with permission, to optimise the response rate. Information sheets with a QR code link to the consent form were re-distributed to psychiatry and clinical psychology professionals until 30 June 2023, subsequently reaching a 40.8% response rate.

### Data collection and analysis

The survey was designed using Research Electronic Data Capture (REDCap), through the University of the Witwatersrand’s REDCap administrator. A self-administered modified 21-item questionnaire used in similar studies was completed online.^12^ Written permission to use and modify the questionnaire for the South African context was obtained from the principal author of the questionnaire. The question ‘ECT is used more often in Hungary than in the USA’ was excluded because of its lack of relevance to the South African context. Additionally, ‘Hungary’ was replaced with ‘South Africa’ in the questions referring to country-specific legislation and practice. All other questions were retained in their original form to preserve the integrity of the questionnaire and facilitate comparison with previous studies that used the same questionnaire.^13,14,15,16,17,18^

Data analysis was done using IBM SPSS Statistics for Windows, Version 28.0 (IBM Corp., Armonk, NY, USA). Demographic data were analysed using descriptive statistics such as mean, median mode and standard deviation. Responses from the questionnaire were compared to determine if any significant differences between the groups existed. Categorical variables were analysed using Chi-squared and continuous variables using the *t*-test for normally distributed variables, and the Mann–Whitney *U* test and Kruskal–Wallis test for variables not satisfying this assumption. The significance level was set at 5%.

### Ethical considerations

Ethical approval was obtained from the University of the Witwatersrand Human Research Ethics Committee (No. M220327). In addition, permission to distribute the questionnaire among clinical psychology and psychiatry professionals was obtained from the University of the Witwatersrand’s Division of Clinical Psychology Head of Department as well as from the Academic Head of the Department of Psychiatry, respectively. Consent for participation was obtained electronically. The invitation emails were sent by the departmental secretaries to all psychiatry and clinical psychology staff email addresses, and no identifying data were used to ensure the anonymity of participants.

## Results

### General characteristics and main sources of knowledge

The survey was completed by 80 psychiatry and clinical psychology professionals at the University of the Witwatersrand. The psychiatry professionals (*n* = 58) constitute 33 psychiatry registrars (41.3%) and 25 psychiatrists (31.3%). The psychology professionals (*n* = 22) constitute 11 clinical psychologists in training (13.8%) and 11 clinical psychologists (13.8%). The median age of the respondents was 33. The majority of respondents were women (*n* = 57, 74.0%). Most respondents rated their knowledge of ECT as medium (*n* = 43, 53.8%) in contrast to minimal (*n* = 26, 32.5%) and high (*n* = 11, 13.8%).

**FIGURE 1:**
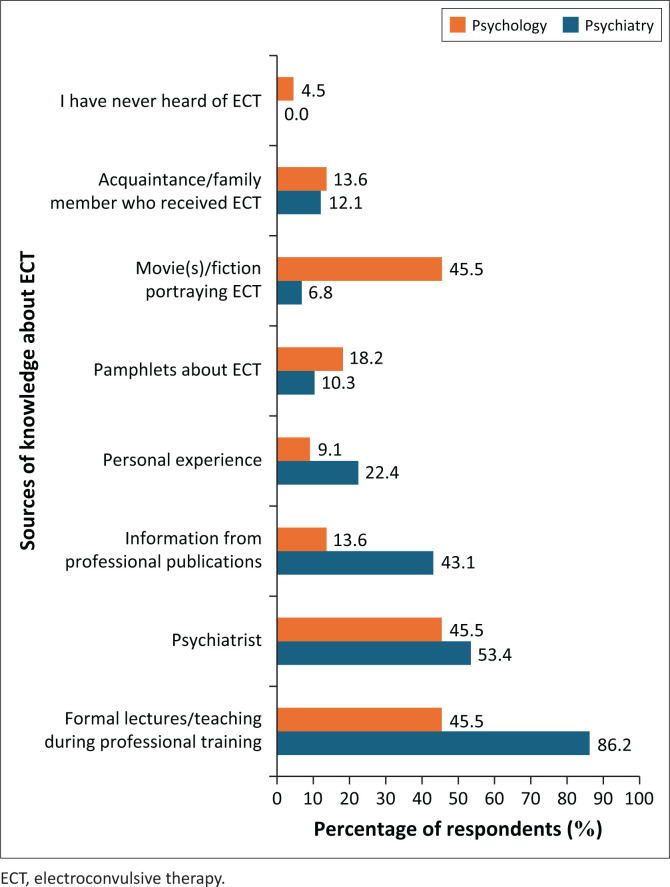
Sources of knowledge of electroconvulsive therapy among psychiatry and psychology professionals.

Most psychiatry professionals (86.2%) obtained knowledge of ECT from formal teaching during their professional training. The most common sources of knowledge on ECT among clinical psychology professionals were formal teaching during training (45.5%) and from psychiatrists (45.5%), with a similar proportion learning about ECT from movies and fiction (45.5%). Sources of knowledge of ECT are represented in Figure 1.

### Exposure to and knowledge of electroconvulsive therapy

Respondents were classified as having had professional exposure to ECT if they worked in an ECT utilising department or had referred patients to this procedure or observed patients receiving it. Most psychiatry professionals (*n* = 54, 93.1%) had professional exposure to ECT compared to 45.5% (*n* = 10) of clinical psychology professionals. Responses to questions regarding knowledge of ECT are presented in Table 1.

**TABLE 1:** Participants’ responses to the knowledge questionnaire.

Questions concerning knowledge about ECT	Psychology (*N* = 22)		Psychiatry (*N* = 58)		Total (*N* = 80)	
	*N*	%	*N*	%	*N*	%
ECT was used for the first time in the 1930s*						
True	17	77.3	52	89.7	69	86.3
False	5	22.7	6	10.3	11	13.8
The anaesthetic level during ECT should be as deep as possible*						
True	7	31.8	18	31	25	31.3
False	15	68.2	40	69	55	68.8
The efficacy of the convulsive treatment has been discovered by a Hungarian psychiatrist*						
True	14	63.6	31	53.4	45	56.3
False	8	36.4	27	46.6	35	43.8
**ECT is more effective and helps to relieve depression faster than drugs do**						
True	12	54.5	48	82.8	60	75.0
False	10	45.6	10	17.2	20	25.0
**ECT is contraindicated in patients with prior history of myocardial infarction**						
True	16	72.7	18	31	34	42.5
False	6	27.3	40	69	46	57.5
**In South Africa, ECT can be administered only under anaesthesia**						
True	15	68.2	55	94.2	70	87.5
False	7	31.8	3	5.8	10	12.5
**ECT can be performed in South Africa without muscle relaxation**						
True	9	40.9	2	3.4	11	13.8
False	13	59.1	56	96.6	69	86.3
**ECT can be used over the age of 65**						
True	5	22.7	45	77.6	50	62.5
False	17	77.3	13	22.4	30	37.5
The longer the seizure duration, the more effective the treatment*						
True	6	27.3	34	58.6	40	50.0
False	16	72.7	24	41.4	40	50.0
**Recommended weekly frequency of the sessions are two or three**						
True	12	54.5	47	81	59	73.8
False	10	45.5	11	19	21	26.3

ECT, Electroconvulsive therapy.

**FIGURE 2:**
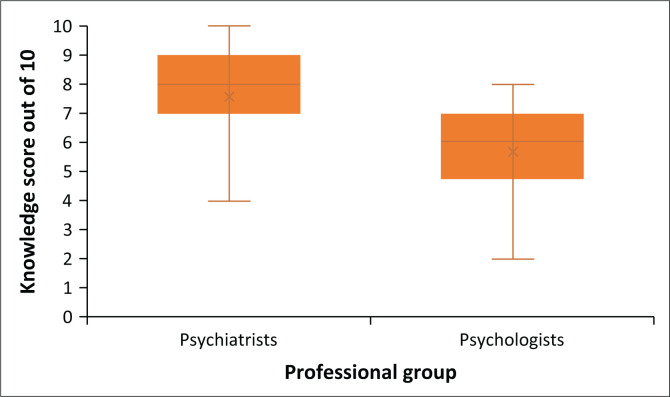
Distribution of the knowledge score obtained by the psychiatry and psychology professional groups.

For the knowledge items, a score of 1 was given for each correct answer and 0 for each incorrect answer. The sum of the scores out of 10 formed the knowledge score of the participant. Psychiatry professionals had a mean knowledge score of 7.6 (± SD1.6) and psychology professionals a score of 5.7 (± SD1.7). The distribution of the knowledge score among the two professional groups is represented in Figure 2.

Both professional groups knew that ECT was used for the first time in the 1930s; however, more clinical psychology professionals (*n* = 14, 63.6%) than psychiatry professionals (*n* = 31, 53.4%) knew that a Hungarian psychiatrist discovered it. Both professional groups indicated correctly on the depth of anaesthesia, but more psychiatry colleagues (*n* = 55, 94.2%) than psychology colleagues (*n* = 15, 68.2%) knew that ECT can only be performed under anaesthesia in South Africa. More psychology professionals (*n* = 9, 40.9%) than psychiatry professionals (*n* = 2, 3.4%) did not know that ECT must be performed with muscle relaxation. On questions related to the contraindications of ECT, most clinical psychology professionals (*n* = 17, 77.3%) did not know that ECT can be used over the age of 65, and the majority (*n* = 16, 72.7%) thought that ECT was contraindicated in patients with prior history of myocardial infarction. Fewer clinical psychologists than psychiatry professionals knew that the recommended frequency of sessions is two to three per week. On the efficacy of ECT, psychiatry professionals (*n* = 48, 82.8%) correctly indicated that ECT helps to relieve depression faster than drugs do. Most psychiatry professionals (*n* = 34, 58.6%) incorrectly indicated that the longer the seizure duration, the more effective the treatment.

### Attitudes towards electroconvulsive therapy

Responses to questions regarding attitudes towards ECT are represented in Table 2. In a similar fashion to a previous study using the same questionnaire, a score of 1 was assigned for responses that indicated a positive attitude and a score of 0 for responses that reflected a negative attitude. Summing the score yielded a total score out of 11; the higher the score, the more positive the attitude.

**TABLE 2:** Participants’ responses to the attitude questionnaire.

Questions concerning attitude toward ECT	Psychology (*N* = 22)		Psychiatry (*N* = 58)		Total (*N* = 80)	
	*N*	%	*N*	%	*N*	%
**Psychiatrists often abuse ECT**						
Agree	0	0	2	3.4	2	2.5
Disagree	22	100	56	96.6	78	97.5
**ECT is used to control violent patients**						
Agree	1	4.5	2	3.4	3	3.8
Disagree	21	95.5	56	96.6	77	96.3
**ECT is used as a punishment**						
Agree	0	0	0	0	0	0
Disagree	22	100	58	100	80	100.0
**ECT can cause pain**						
Agree	14	63.6	32	55.2	46	57.5
Disagree	8	36.4	26	44.8	34	42.5
**ECT is dangerous and may cause death**						
Agree	6	27.3	5	8.6	11	13.8
Disagree	16	72.7	53	91.4	69	86.3
**ECT is used more often for treating poor people**						
Agree	0	0	1	1.7	1	1.3
Disagree	22	100	57	98.3	79	98.8
**ECT should only be used as a last resort**						
Agree	14	63.6	14	24.1	28	35.0
Disagree	8	36.4	44	75.9	52	65.0
**ECT is used more often in populations that access the public health sector**						
Agree	6	27.3	8	13.8	14	17.5
Disagree	16	72.7	49	84.5	65	81.3
Missing	0	0	1	1.7	1	1.3
**ECT is an outdated, obsolete procedure**						
Agree	3	13.6	1	1.7	4	5.0
Disagree	19	86.4	56	96.6	75	93.8
Missing	0	0	1	1.7	1	1.3
**ECT can cause permanent brain damage**						
Agree	10	45.5	7	12.1	17	21.3
Disagree	12	54.5	49	84.5	61	76.3
Missing	0	0	2	3.4	2	2.5
**ECT should be illegal to perform**						
Agree	2	9.1	0	0	2	2.5
Disagree	19	86.4	57	98.3	76	95.0
Missing	1	4.5	1	1.7	2	2.5

ECT, Electroconvulsive therapy.

**FIGURE 3:**
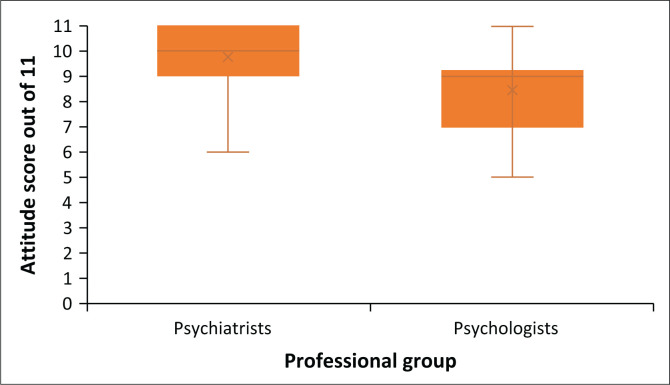
Distribution of the attitude score obtained by the psychiatry and psychology professional groups.

Psychiatry professionals obtained a mean attitude score of 9.8 (± s.d. 1.2), whereas psychology professionals obtained a score of 8.5 (± s.d. 1.8), indicating a trend towards a positive attitude towards ECT among both psychiatry and psychology professionals. The distribution of the attitude score among the two professional groups is represented in Figure 3.

All respondents disagreed that ECT is used as punishment (*n* = 80, 100%). While all clinical psychology professionals did not believe that psychiatrists often abuse ECT (*n* = 22, 100%), a small number of psychiatrists (*n* = 2, 3.4%) did. Similar to most psychiatry professionals (*n* = 56, 96.6%), most psychology professionals (*n* = 21, 95.5%) did not agree that ECT is used to control violent patients (*n* = 77, 96.3%). There were mixed perceptions relating to the side effects of the procedure, with 63.6% (*n* = 14) of clinical psychologists and 55.2% (*n* = 32) of psychiatry professionals indicating that ECT can cause pain. Almost half of psychology professionals (*n* = 10, 45.5%) believed that ECT can cause permanent brain damage, whereas a large majority of psychiatry professionals (*n* = 49, 84.5%) did not agree. Most respondents from both professional groups did not perceive ECT as dangerous or potentially fatal, with 86.3% (*n* = 69) expressing this view. Regarding the application of ECT, a majority of clinical psychology professionals (63.6%, *n* = 14) indicated that it should be considered a last resort, whereas a higher proportion of psychiatry professionals (75.9%, *n* = 44) did not share this view. None of the respondents (100%, *n* = 80) believed that ECT is disproportionately used to treat individuals from lower socioeconomic backgrounds. Additionally, the majority of psychiatry professionals (84.5%, *n* = 49) and clinical psychology professionals (72.7%, *n* = 16) did not believe that ECT is more frequently administered in the public sector. A significant majority from both professional groups disagreed with the notion that ECT is outdated and obsolete (93.8%, *n* = 75) and that it should be deemed illegal (95%, *n* = 76).

### Relationship between electroconvulsive therapy knowledge score and attitude towards electroconvulsive therapy

The Mann–Whitney *U* hypothesis test was used to compare the knowledge scores and the attitude scores, which showed a significant difference for both the knowledge score *p* < 0.001 and the attitude score *p* < 0.001 between the two professional groups. Furthermore, there was a significant association between the knowledge score and the attitude score (*p* = 0.01), with an odds ratio (OR) of 6.7 (Chi-Squared).

There was no significant difference in the knowledge scores (*p* = 0.31) and attitude scores (*p* = 0.62) between male and female professionals. Similarly, the age difference was not significant for both the knowledge score (*p* = 0.27) and the attitude score (*p* = 0.26). The difference in the knowledge score (*p* = 0.049) and attitude score (*p* = 0.01) between trainees, that is, psychiatry registrars and clinical psychology interns/community service was significant. Conversely, the difference in the attitude score was significant (*p* = 0.01) among trainee professionals whereas not significant for consultant psychiatrists and registered clinical psychologists.

Overall, most participants (*n* = 62, 77.5%) indicated that they would consent to receive ECT if they were in a psychotic depressive condition, of which most (*n* = 52, 89.7%) were psychiatry professionals and fewer were psychology professionals (*n* = 10, 45.5%). The majority (*n* = 54, 87.1%) of the participants who would consent to ECT had professional exposure to ECT. Consent to receive ECT if they had a depressive episode was associated with a higher knowledge of ECT than those who would not consent to ECT (*p* = 0.03, Mann–Whitney *U* test), and similarly a more favourable attitude towards ECT than those who would not consent to ECT (*p* < 0.001).

All psychiatry professionals (*n* = 58, 100%) and a high proportion of clinical psychology professionals (*n* = 20, 90.9%) would recommend that ECT teaching should be a routine part of psychiatric specialisation. With respect to theoretical ECT training being part of psychology training, a larger proportion of clinical psychology professionals (*n* = 19, 86.4%) than psychiatry professionals (*n* = 39, 67.2%) would recommend its inclusion in the training of clinical psychologists. The associations with knowledge and attitude scores are summarised in Table 3.

**TABLE 3:** Associations with knowledge and attitude scores.

Variable	*P*		Statistical test
	Knowledge	Attitude	
Age (categorical)	0.270	0.260	Kruskal-Wallis hypothesis test
Gender (male/female)	0.310	0.620	Mann-Whitney U
Would consent to ECT (yes/no)	0.030	< 0.001	Mann-Whitney U
Exposure to ECT (professional/personal/none)	0.005	0.009	Kruskal-Wallis hypothesis test

ECT, Electroconvulsive therapy.

Four potentially irrelevant or ambiguous questions on the knowledge questionnaire (Marked with asterisk in Table 1) were removed to better assess the relationship between knowledge and attitude towards ECT. Reanalysis showed consistent patterns and did not alter the study’s findings. Significant differences were observed between knowledge scores of the two professional groups (*p* < 0.001) and between knowledge and attitude scores (*p* = 0.002), with an OR of 7.3 (Chi-Squared). No significant differences were found based on gender (*p* = 0.44) or age (*p* = 0.139). Additionally, individuals who would consent to ECT demonstrated greater knowledge about ECT compared to those who did not consent (*p* = 0.005).

### Concerns regarding electroconvulsive therapy

**FIGURE 4:**
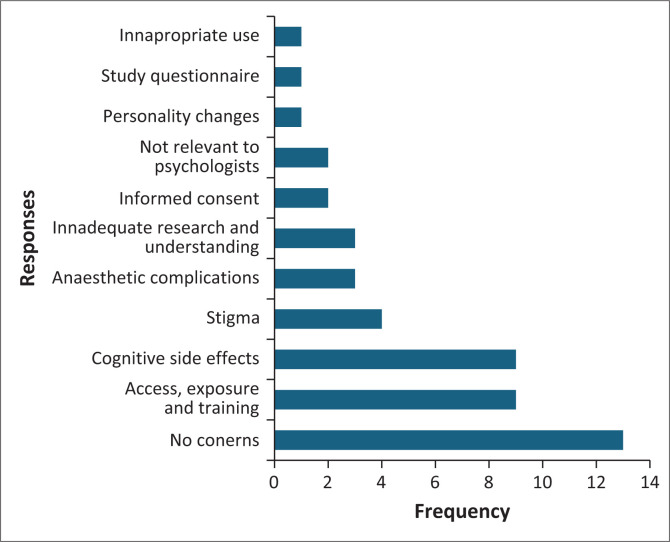
Open ended question on concerns regarding electroconvulsive therapy.

Respondents were additionally asked to identify and elaborate on their concerns related to ECT; 43 of the 80 participants chose to respond to this section of the questionnaire. A summary of the responses is displayed in Figure 4. In all, 13 participants specified having no concerns about ECT. Concerns on the availability of ECT in institutions, the exposure of psychology professionals to this treatment and the adequacy of training for psychiatry professionals administering ECT were raised. Concerns with cognitive side effects (*n* = 9) such as memory loss and other cognitive impairments, anaesthetic side effects (*n* = 3) and personality changes (*n* = 1) were also noted. The stigma associated with ECT also emerged as a concern (*n* = 4). There were concerns regarding the level of research and understanding related to ECT (*n* = 3). Respondents felt that there was insufficient scientific investigation and public knowledge about the procedure, which could affect its acceptance and application. The issue of informed consent was raised (*n* = 2), with respondents concerned about whether patients fully understand the risks and benefits of ECT before consenting to the treatment. A minority of respondents (*n* = 2) questioned the relevance of ECT in the field of psychology. One respondent noted concerns about the inappropriate use of ECT, indicating a need for stricter guidelines and oversight in its application. There was a concern (*n* = 1) regarding the validity of the study questionnaire itself.

## Discussion

The equal proportions of clinical psychology professionals citing movies and fiction, psychiatrist and formal teaching as sources of knowledge suggest that public portrayals of ECT may shape their understanding and attitudes towards the therapy. In South Africa, medical students similarly relied on media rather than professional sources, leading to mixed perceptions of ECT as a last resort.^19^ Notably, 93.1% of psychiatry professionals reported direct exposure to ECT through clinical practice or patient referrals, compared to only 45.5% of clinical psychology professionals. This disparity highlights the impact of direct professional experience on ECT knowledge and competency, as reflected in the higher mean knowledge score for psychiatry professionals (7.6 out of 10, ± s.d. 1.6) compared to clinical psychology professionals (5.7 out of 10, ± s.d. 1.7). In a study done in Israel, psychiatrists and nurses were shown to have a more positive attitude than other mental health professionals, attributing it to a greater exposure to ECT.^20^

Both groups were aware that ECT was first introduced in the 1930s, with clinical psychology professionals demonstrating slightly better knowledge of its Hungarian origins (63.6%) compared to psychiatry professionals (53.4%). This minor difference might reflect variations in historical education or interest between the disciplines. However, given the higher mean knowledge score among psychiatrists, this specific question may not fully capture core ECT knowledge. Significant differences were observed in procedural knowledge: while both groups recognised the need for anaesthesia during ECT, a larger proportion of psychiatry professionals (94.2%) were aware that ECT must be administered under anaesthesia compared to clinical psychologists (68.2%). Additionally, fewer clinical psychologists (40.9%) were aware of the need for muscle relaxation during ECT. Knowledge gaps among clinical psychology professionals concerning contraindications, such as ECT’s applicability for patients over 65 (77.3%) and those with a history of myocardial infarction (72.7%), could affect clinical decision-making. On treatment efficacy, psychiatry professionals were better informed that ECT offers faster relief from depression compared to pharmacotherapy (82.8%). However, 58.6% of psychiatry professionals incorrectly believed that longer seizure duration correlates with greater treatment effectiveness, which may be because of the ambiguous nature of this question regarding what constitutes a long seizure duration.

The unanimous agreement that ECT is not used as punishment (100%) indicates a shared recognition of ECT’s therapeutic intent across both professional groups. This consensus is reinforced by the fact that no clinical psychology professionals believed that psychiatrists frequently misuse ECT, although a small minority of psychiatrists (3.4%) expressed concerns about potential misuse. Both groups largely agree that ECT is not used to control violent patients, with high levels of disagreement on this notion (96.3%).

Perceptions about the side effects of ECT reveal some discrepancies between the professional groups. While a majority in both groups reject the notion that ECT is inherently dangerous or potentially fatal (86.3%), concerns about pain and potential long-term effects are notable. The belief that ECT can cause permanent brain damage was held by 45.5% of clinical psychologists compared to 15.5% of psychiatrists. The lower incidence of such beliefs among psychiatrists suggests they may have more experience with ECT’s safety profile. Views on the role of ECT as a treatment option vary between the groups. A majority of clinical psychology professionals (63.6%) consider ECT a last-resort treatment, while a higher proportion of psychiatrists (75.9%) do not share this view. This finding contrasts with international studies, which have shown that a significant proportion of psychiatrists and other mental health professionals believe ECT should be reserved as a last-resort treatment.^13,14,15,20^ This disparity may also indicate differing thresholds for recommending ECT based on professional roles. Furthermore, the strong rejection of views that ECT is outdated or should be deemed illegal (93.8% and 95.0%, respectively) signifies a broad endorsement of its continued relevance in contemporary psychiatric practice.

The significant association between knowledge and attitude scores (*p* = 0.01) with an OR of 6.7 suggests that higher knowledge of ECT is related to more positive attitudes towards the treatment. This supports earlier studies correlating positive attitudes towards ECT with clinical experience and knowledge of ECT.^6,7,13,14,15,16,17,18,19,20^ The correlation indicates that efforts to increase knowledge among clinical psychology professionals could also improve their attitudes towards ECT. A similar finding was made among medical students in Nigeria, who demonstrated a better attitude towards ECT after clinical exposure to ECT and lectures.^21,22^

The majority of responses to the question on concerns about ECT expressed ‘no concerns’. This may reflect familiarity with ECT’s benefits and trust in its clinical efficacy. A high proportion of the responses expressed concerns related to the availability of ECT, the exposure of psychology professionals to this treatment and the adequacy of training for psychiatry professionals administering ECT. These concerns highlight systemic issues that could impact the accessibility of ECT services. In a review of the use of ECT within South Africa, Benson-Martin noted that although 42 institutions in the public and private sectors had access to an ECT machine, 31% did not use the machine because of various problems including clinicians not agreeing with the procedure.^8^

A similar proportion raised concerns about cognitive side effects. Cognitive impairments are well-documented side effects of ECT, though their severity and impact on patients’ quality of life can vary.^23^ This concern reflects a need for clinical strategies to shed light on the chronicity and impact of cognitive side effects on patient quality of life. Anaesthetic side effects were less emphasised compared to cognitive effects, but they remain an important consideration in the overall safety profile of ECT. The mention of personality changes by one respondent, while not supported by empirical evidence as a direct side effect of ECT, reflects a possible concern about potential long-term psychological impacts.

Some responses expressed concerns about the stigma associated with ECT, which may arise from its historical misuse and media portrayal. There is a call for more research to better understand ECT’s mechanism, efficacy and long-term effects. Additionally, there was concern that patients might not fully grasp the risks and benefits of ECT, suggesting a need for improved educational resources and counselling. Questions about ECT’s relevance in psychology versus psychiatry indicate differing views on its role. Finally, issues with wording of the questionnaire highlight the need for precise tools to accurately assess knowledge and attitudes towards ECT.

### Strengths and limitations

Several limitations should be acknowledged in interpreting the findings of this study. Firstly, the sample size was relatively small with a total response rate of 40.8%, consisting of professionals from a single institution, which may limit the generalisability of the results to broader populations of psychiatry and clinical psychology professionals. Secondly, the survey relied on self-reported data, which may be subject to recall bias and social desirability bias. Thirdly, the survey instrument itself may not have captured all relevant aspects of knowledge and attitudes towards ECT, potentially leading to incomplete or biased responses. The wording of some of the questions in the questionnaire may have been ambiguous, interfering with the interpretation of knowledge and attitude scores. Lastly, the clinical psychology and psychiatry professional groups are not homogenous groups of professionals as there may be differences in the years of experience and exposure to ECT.

### Recommendations

Based on the findings and limitations identified in this study, several recommendations can be made to inform future research and practice in this area. Firstly, larger-scale studies involving diverse samples of mental health professionals from multiple institutions are needed to enhance the generalisability of findings and ensure representation across different contexts. Secondly, future research should utilise more comprehensive and validated measures of knowledge and attitudes towards ECT to obtain a more accurate understanding of professionals’ knowledge and perceptions. Qualitative studies could explore the underlying themes behind any reported negative perceptions, providing a richer and more nuanced understanding of these attitudes. For psychiatry professionals, continued emphasis on formal teaching remains essential, including the integration of current research. Interdisciplinary collaboration and training initiatives could further enhance cooperation between psychiatry and psychology professionals in the delivery of mental healthcare, including education and exposure to ECT.

## Conclusion

This research provides insights into the knowledge and attitudes towards ECT among psychiatry and clinical psychology professionals. Despite differences in levels of knowledge, both groups generally expressed positive attitudes towards ECT, highlighting its perceived efficacy in treating certain psychiatric disorders. Because of the association between knowledge of and attitude towards ECT, addressing gaps in knowledge and promoting evidence-based practice are essential for ensuring the appropriate use of the procedure and improving patient outcomes. Moving forward, collaborative efforts between psychiatry and psychology professionals, coupled with targeted education and training interventions, are crucial for advancing the field of ECT and enhancing mental healthcare delivery.
